# Congenital syphilis as the cause of multiple bone fractures in a young infant case report

**DOI:** 10.1186/s12887-022-03789-y

**Published:** 2022-12-21

**Authors:** Maria Koliou, Elpida Chatzicharalampous, Myria Charalambous, Kyriakos Aristeidou

**Affiliations:** 1 Medical School, University of Cyprus, Palaios Dromos Lefkosias- Lemesou, 5 Agiou Symeon Street, Strovolos, Nicosia, 2037 Cyprus; 2 Department of Paediatrics, Archbishop Makarios III Hospital, 6 Korytsas Str, 2012 Nicosia, Cyprus; 3 Department of Radiology, Archbishop Makarios III Hospital, 6 Korytsas Str, 2012 Nicosia, Cyprus

**Keywords:** Bone fractures, Infant, Hepatosplenomegaly, Congenital syphilis, Case report

## Abstract

**Background:**

The differential diagnosis of multiple unexplained bone fractures in a young infant usually includes child abuse or bone disease such as osteogenesis imperfecta. Bone abnormalities can occur in 60–80% of cases with congenital syphilis and may be the sole manifestation. However, this frequent manifestation of this rare disease such as congenital syphilis is frequently disregarded. We describe a case of a young infant with multiple long bone fractures diagnosed with congenital syphilis.

**Case presentation:**

This 2-month-old male patient was referred to our hospital because of fractures of the ulna and distal radius bilaterally and noisy breathing with the suspicion of osteogenesis imperfecta. After thorough examination, the infant had anemia and a palpable spleen. We performed a screen for congenital infections among other investigations, which revealed positive non-treponemal and treponemal antibodies for syphilis. Hence the diagnosis for Congenital Syphilis was made.

We performed a lumbar puncture (LP) which showed mild pleocytosis. The patient was treated with intravenous aqueous penicillin G 200 000 UI/KG per day for 10 days. In addition, a single dose of intramuscular penicillin G benzathine 50 000 UI/KG was given due to the abnormal result of CSF.

On follow up admission 6 months later, the new syphilis serology had much improved and the new LP revealed no abnormal findings.

**Conclusions:**

We present this case report in order to remind of a common manifestation of congenital syphilis, a rare disease which needs to be included in the differential diagnosis of multiple unexplained fractures in early infancy. In our case the fractures were symmetric and bilateral and they were accompanied by anemia and mild hepatosplenomegaly which led to the investigation of congenital syphilis as a possible cause. However, two thirds of infants with congenital syphilis are asymptomatic at birth. All women should have a proper syphilis screening during pregnancy.

## Background

Syphilis is caused by spirochete *Treponema pallidum*. Vertical transmission of *Treponema pallidum* in pregnant women who are inadequately treated or not treated at all can lead to congenital infection, causing various manifestations. Up to 40% of babies born to women with untreated syphilis are stillborn; other immediate complications include hydrops fetalis, preterm birth, low birthweight or may be asymptomatic at birth [1]. Infected infants can have severe anemia, jaundice, hepatosplenomegaly, snuffles, mucocutaneous lesions, pneumonia, osteochondritis, periostitis, pseudoparalysis at birth or within 4 to 8 weeks after birth [2].

Some children remain asymptomatic for years with neurological complications only becoming apparent later in life. Early diagnosis and treatment are important because late manifestations can be prevented by treatment of early infection. Early recognition of congenital syphilis might be delayed if physicians do not consider it as a possible diagnosis in a baby with bone fractures.

According to the World Health Organization (WHO), the incidence of congenital syphilis has decreased worldwide especially between 2012 and 2016 [3]. However, it appears that the rate of congenital syphilis varies in different continents. In the US the rate of congenital syphilis reported has dramatically increased between 2012 and 2019 as the 2019 national rate of 48.5 per 100,000 live births represented a 477% increase in comparison to 2012 (8.4 cases per 100,000 live births) [4]. On the other hand, in European Union/European Economic Area (EU/EEA) countries the rate of congenital syphilis appears to have decreased from 2.5 cases per 100,000 live births in 2009 to 1.6 per 100,000 live births in 2018. This means only 60 confirmed cases of congenital syphilis in all 23 EU/EEA Member states in 2018. However, it is perceived that a substantial percentage of cases of congenital syphilis is underreported [5].

The differential diagnosis of multiple unexplained fractures in early infancy may be challenging. The most frequent cause of multiple fractures at this age is child abuse, however bone disease associated with osteopenia and increased fragility has also to be excluded [6–10].

In our case the child’s initial suspicion by the Paediatric Orthopaedic Surgeon was osteogenesis imperfecta and was finally diagnosed as congenital syphilis. This case report is presented in order to remind of a common manifestation of a rare disease which needs to be included in the differential diagnosis of multiple unexplained fractures in early infancy. It also stresses the importance of screening for syphilis during pregnancy as most of the cases in infants can be prevented if the mother is diagnosed and treated early.

## Case presentation

**Fig. 1:**
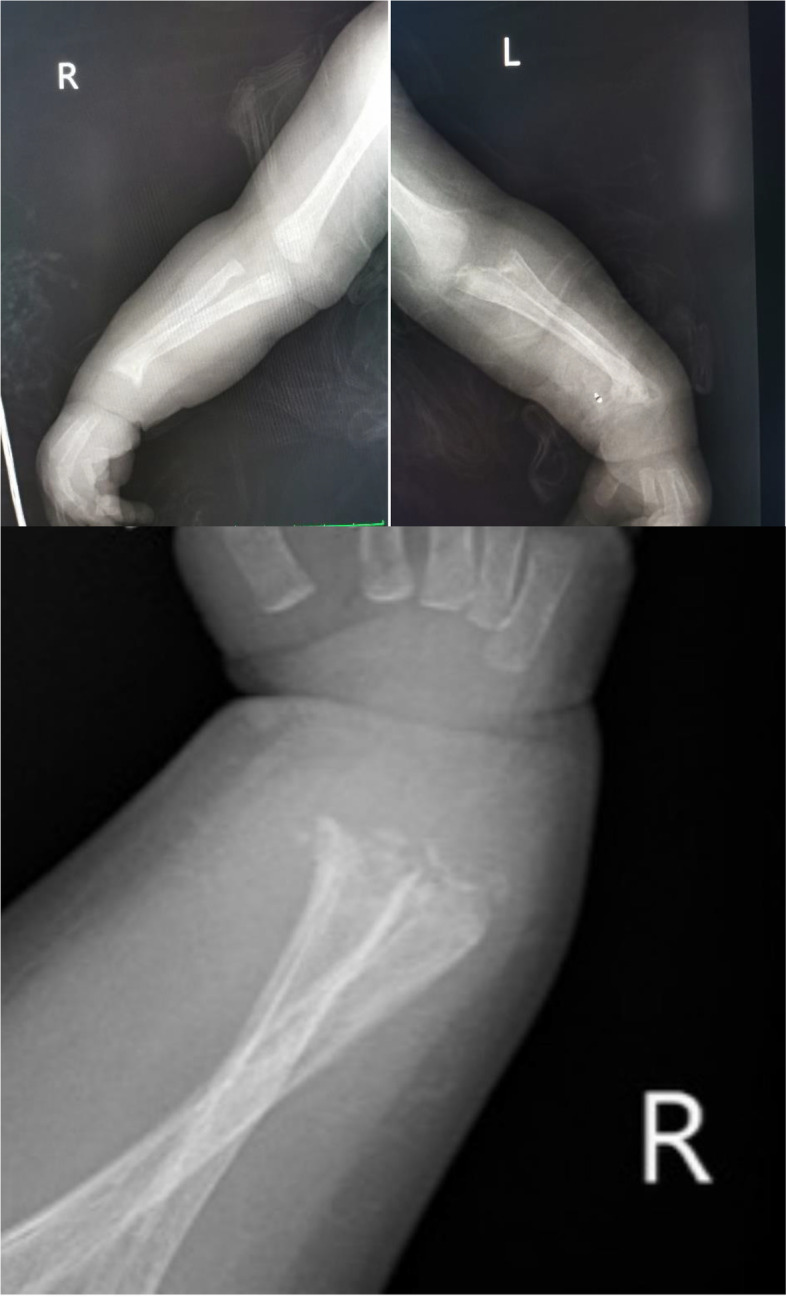
X-ray findings of long bones

A previously healthy 2-month old male infant with parents from Romania and Lebanon presented to our hospital for investigation of fractures of the ulna and distal radius bilaterally. In addition, the infant had noisy breathing and signs of runny nose and reduced feeding. A few days earlier the patient’s mother had noticed that the patient couldn’t move his upper extremities and started to cry whenever she tried to passively move them. The day before admission the child was examined by an Orthopedic surgeon who asked for an x ray of the upper limbs which revealed a supracondylar fracture of the left elbow as well as lytic areas on the distal radius and ulna of both hands with probable fracture and displacement. Mild osteopenia in some areas was also noted. In addition, the end plates of peripheral radius and ulna bilaterally appeared with distorted edge (Fig. 1). The child was referred to the Genetics clinic for further investigation for possible osteogenesis imperfecta (OI). The child was admitted to the Paediatric ward for all diagnostic work up.

On admission the child was well in himself, with mild hypotonia, hypertelorism and low base of the nose. He had mild tachypnoea with rhinitis and palpable liver and spleen.

He was born after a full-term uneventful pregnancy delivered by caesarian section, birth weight 3.020 kg. No problems in the perinatal period. He was gaining weight well. No family history of metabolic or genetic disease.

Laboratory workup revealed anemia, hyponatremia, low albumin, raised globulin, increased alanine aminotransferase (ALT), aspartate aminotransferase (AST) and D-dimers (DD).

### Laboratory investigations in detail

Hemoglobin (Hb) of 7 g/dL, white blood cell (WBC) count of 14 × 10^3^ cells/μL (35% segmented neutrophils, 52% Lymphocytes, 9% Monocytes, 2% eosinophils), platelet count of 421 × 10^3^ /μl, sodium of 130 mmol/L, albumin 3 g/dl (3.7–4.4), globulin 4.7 g/dl (1.5–3.5), AST of 79 U/L, ALT of 142 U/L, ferritin 240 ng/ml (10–160), alkaline phosphatase (ALP) 431U/L (82–383) and C-reactive protein (CRP) of 47 mg/L(neg < 0.5). Serum Calcium, Parathyroid hormone (PTH), Vitamin D (25-OH Vitamin D), Coagulation and Thyroid hormones were within normal levels. Furthermore, we performed brain ultrasound, brain MRI, chest x ray, fundoscopy and hearing test which revealed no abnormal findings. A skeletal survey showed no other fractures except the above. Basic Metabolic Panel (BMP), molecular and simple karyotype were normal. Urine Biochemistry was also normal.

Abdominal ultrasound showed mild splenomegaly 6.8 cm (normal dimensions 4.8 ± 0.5 cm) and mild hepatomegaly 7 cm (6 cm ± 0.5 cm). Because of the mild hepatosplenomegaly we decided to screen for congenital infections: The screening for Toxoplasma, Rubella, parvovirus B19, Cytomegalovirus, Herpes Simplex virus and HIV were all negative. The syphilis screening resulted in positive non-treponemal and treponemal antibodies as shown in Table 1. Hence, the diagnosis for Congenital Syphilis was made.

**Table 1:** Syphilis antibody results

**Laboratory method**	**On first admission**	**On 6-month admission**
	**Result**	**Result**
Rapid Plasma Reagin (RPR)	1/64	1/4
Treponema pallidum Hemagglutination assay (TPHA)	> 1:2560	> 1:2560
Treponema pallidum Total IgG/IgM	positive	Positive
Treponema pallidum IgM	positive	Indeterminate

We performed a lumbar puncture (LP) (Table 2) which revealed increased white blood cell count, normal glucose level and slightly raised protein level. Western Blot for syphilis was positive. Cerebrospinal Fluid (CSF) culture was negative.

**Table 2:** Cerebrospinal fluid findings upon admission and at 6-month visit

	**On first Admission**	**On 6-month admission**
CSF-white blood cell count	12/μL	1/μL
CSF-red blood cell	105/μL	1/μL
CSF-glucose	48 mg/dL (blood gluc 99 mg/dl)	60 mg/dL
CSF-protein	48 mg/dL	30 mg/dL
CSF-VDRL	–-	Negative
Western blot	positive	Very low positive

The patient was transfused with packed red blood cells and was treated for congenital syphilis which involved the Central nervous system (CNS) with intravenous aqueous penicillin G (50.000 UI/kg Q4hr for 10 days). A single dose of intramuscular penicillin G benzathine (50 000 UI/kg), was also given. The patient was discharged and a scheduled follow up readmission after 6 months was arranged.

On his 6-month follow up admission at the age of 9 months, the child was very well in himself with normal psychomotor development for his age.

The LP was repeated at this admission and the results are shown in Table 2. All findings were within normal levels except a very low positive Western blot test.

A new syphilis serology revealed Non-treponemal test (RPR) significantly lower (four times) than the previous admission results (Table 1). The patient was discharged with instructions for subsequent follow-up by the pediatric infectious disease specialist in 3 months’ time.

When the child was diagnosed with congenital syphilis, the parents were tested for syphilis and were found positive. Therefore, they were referred to their General Practitioner for treatment.

## Discussion

Published series on long bone fractures in young infants usually suggest differential diagnosis between child abuse, osteogenesis imperfecta and other bone diseases associated with increased bone fragility [8, 11]. Congenital syphilis is one of the rare causes of this finding which can well be associated with demineralization and abnormalities of bones resulting in pathologic fractures or pain which may limit movement of the limb. On the other hand, abnormal long bone x rays, are a frequent manifestation of early congenital syphilis, as this may be found in 60 to 80% of cases of babies born by mothers with untreated syphilis in pregnancy [12, 13].

Osteogenesis imperfecta (OI) is usually caused by different mutations of one of the two genes COLIA1 and COLIA2 which encode the chains of type 1 collagen, the major protein constituent of bone [14]. The diagnosis of OI in many patients can be based on diagnostic signs such as osteopenia, bone deformities and characteristic appearance of the bones of the scull (wormian bones) in x ray investigation [15]. However, its phenotypic expression can be quite heterogeneous and the diagnostic signs may be subtle. A careful medical and family history may also be helpful although in some cases family history of bone disease may be absent as spontaneous mutations are also common [6].

In our case the guiding sign was the mild hepatosplenomegaly which made us investigate for congenital infections one of which was syphilis. The child also had anemia, hyponatremia, low albumin and high globulin, slightly raised liver enzymes and alkaline phosphatase probably because of the fractures. Anemia is also a common finding compatible with congenital syphilis. Anemia developing after the neonatal period is usually non-hemolytic as it was in this case report. Rhinitis usually develops during the first week of life or less frequently during the first 3 months of life. It is usually persisting. In our case it was not clear from the medical history when it had started as the mother could not remember. It was one of the features present during the child’s hospitalization which means it was present during the second month of life. Rhinitis was the cause of mild tachypnoea and difficulty in breathing in the two-month-old infant. Nasal discharge usually contains spirochetes and is contagious. The patient had also involvement of the central nervous system as shown by the CSF findings of increased white blood cell content. Normal levels of vitamin D and non-typical skeletal abnormalities also excluded rickets in our case.

The findings on the long bones in this case report were characteristically symmetric with lytic areas and fractures on both radius and ulna bilaterally. Further to this, the end plate of peripheral radius and ulna appeared with distorted edge. Such symmetric polyostotic abnormalities affecting the long bones are the most frequently observed findings in CS. Large series of cases were reported before 2000. Later, because of the rarity of this disease, it was mainly case reports that were published in the literature [16, 17].

In 1931 McLean published a series of 102 patients where he described three main types of skeletal anomalies ie osteochondritis, periostitis, osteomyelitis and more rarely osteitis [18]. In 1953 Engeset, Eek and Gilje in their study of 59 cases, suggested that skeletal changes were the result of disturbance in bone growth instead of inflammation [19]. Similarly, Cremin and Fisher in 1970, in their report of 102 cases of CS from South Africa, considered their skeletal findings not due to an active inflammation of the bones but dystrophic in nature instead [20]. Therefore, they classified their findings into three different groups ie Group I: Metaphyseal dystrophy, Group II: Osteitis-like dystrophy and Group III: Periosteal dystrophy.

When later Sachdev M in 1982 described a series of 55 cases from India, the most frequent osseous findings were metaphysitis, zone of rarefaction, periostitis, disorganized metaphysis, bone erosion and the Wimberger’s sign. The main bones affected were the long bones radius, ulna, tibia, femur, humerus and fibula [21].

Later in 1989, in Rasool MN’s series of 197 cases from S Africa the most frequent findings included diaphysitis with periosteal reaction and osteitis, metaphysitis with dense, lucent and alternating bands, combined lesions, dactylitis and pathological fractures as well as joint involvement [22].

After 1989, isolated case reports have mainly been published because of the rarity of the disease. In these reports, similar lesions were reported as the ones described in the previously mentioned series [9, 10, 16, 17]. The main message of these reports was to alert physicians on the possibility of CS in cases of fractures in young infants as CS was becoming more rare as a diagnostic possibility.

Congenital syphilis is very rare in Cyprus. According to the ECDC Annual epidemiological report for 2018, Cyprus reported no cases of congenital syphilis between 2014 and 2018 [5].

Syphilis screening in pregnant women during their antenatal care, is practiced in most pregnancies in Cyprus. As there are no local guidelines for syphilis screening in the country, physicians in Cyprus usually follow guidelines issued by International authorities such as the Public Health England and the Center for Disease Control and prevention [23, 24]. However, it seems that not all pregnant women are properly screened during pregnancy.

The early recognition and treatment of early syphilis in an infant is sometimes challenging especially because around two-thirds of liveborn newborns are asymptomatic at birth [25]. Pediatricians as well as radiologists should become more familiar with common manifestations of these rare diseases as the epidemiology of these diseases is changing. The finding of bone fractures on this baby made the physicians who examined the child before admission, initially consider the possibility of genetic bone disease such as osteogenesis imperfecta. Child abuse might have been another possible option as it is considered the most frequent cause of multiple bone fractures in infants and toddlers [6]. However, the concurrent findings of hepatosplenomegaly and anemia in our case, turned to the possibility of congenital infections one of which was syphilis.

The universal implementation of antenatal syphilis screening programs has proved to be cost effective in both high income and low-income countries [26, 27]. Early detection and treatment of disease in pregnant women almost entirely prevents congenital syphilis in the offspring [25]. In the US, the Centers for Disease Control and Prevention, the American Academy of Pediatrics and the American College of Obstetrics and Gynecologists recommend universal first trimester screening for syphilis and additional third trimester screening for pregnant women with high risk behaviour for syphilis [28–30]. In an ECDC survey in 2013 it was found that all participating EU/EEA countries (26/26) implemented antenatal screening programs for syphilis in the first trimester of pregnancy and some of them also in the third trimester [31].

## Conclusion

This case report reveals the importance of congenital syphilis, despite being a rare disease, to be included in the differential diagnosis of long bone fractures in very young infants, especially if accompanying symptoms or findings are present such as hepatosplenomegaly or anemia. CS can cause demineralization of long bones resulting in pathologic fractures in young infants. Different types of abnormalities on long bones x rays are quite frequent in early CS occurring in around 60–80%. These long bone abnormalities are usually bilateral, symmetric and polyostotic. If anemia presents after the neonatal period it is usually non- hemolytic [2].

As the epidemiology of Congenital syphilis is changing globally, pediatricians should be more familiar to the manifestations of congenital syphilis in young infants and should include congenital syphilis in the differential diagnosis of multiple bone fractures in this age group. Radiologists should also be able to recognize these characteristic long bone abnormalities and guide the pediatricians to the suspicion of congenital syphilis. Furthermore, Obstetricians should get familiar with the screening algorithms and screen every pregnant woman for syphilis in the first trimester of pregnancy and if necessary, ie in women with high risk behaviors, rescreen them in the third trimester according to International guidelines [23, 24].

## Data Availability

N/A as all data are included in this published case report.

## Abbreviations

ALP: Alkaline Phosphatase
ALT: Alanine Aminotransferase
AST: Aspartate aminotransferase
BMP: Basic Metabolic Panel
CM: Centimeter
CNS: Central Nervous System
CRP: C-reactive protein
CS: Congenital Syphilis
CSF: Cerebrospinal Fluid
DD: Ddimers
ECDC: European Centre for Disease Prevention and Control
EU/EEA: European Union/ European Economic Area
GP: General practitioner
Hb: Hemoglobin
HIV: Human Immunodeficiency virus
IgG: Immunoglobulin G
IgM: Immunoglobulin M
Kg: Kilogram
LP: Lumbar Puncture
MRI: Magnetic Resonance Imaging
OI: Osteogenesis imperfecta
PTH: Parathyroid Hormone
RPR: Rapid Plasma Reagin
STI: Sexually transmitted infection
TPHA: Treponema pallidum Hemagglutination assay
UI: Units
VDRL: Venereal Disease Research Laboratory
WBC: White Blood Cell
WHO: World Health Organization
